# Supplementary material for: Content-rich biological network constructed by mining PubMed abstracts
Source: BMC Bioinformatics. 2004 Oct 8;5:147. doi: 10.1186/1471-2105-5-147 (PMC528731; doi:10.1186/1471-2105-5-147)
Supplement: Additional File 2 — The original results of the above study (non-essential files are deleted to keep the file size under the limit set by BMC bioinformatics). [file 1471-2105-5-147-S2.bz2 › chilibotAdditionalFile2/dip50/579ID9437425E10947/html/CHEY_CHEA.html]

 


 **CHEY** and **CHEA** 
  
Found 134 abstracts in PubMed, retrieved 50.  
 

 What does Google say? 
 PDF only 
| .edu only 

---

**Interactive relationship** (e.g. stimulation, inhibition, etc)

**Neutral relationship**- The CheZ binding surface of  **CheY**  overlaps the  **CheA**  and FliM binding surfaces.  Ref: 9295320 J Biol Chem, 1997
- These interactions could entail inactivation of multiple receptor  **CheA**  signaling complexes and or simultaneous activation of  **CheY**  P dephosphorylation.  Ref: 10500179 Proc Natl Acad Sci U S A, 1999
- **CheA**   **CheY**  phosphotransfer generates phospho  **CheY** ,  **CheY**  P.  Ref: 11243808 J Mol Biol, 2001
- Autophosphorylated  **CheA**  passes its phosphoryl group to  **CheY**  very rapidly k cat approximately 750 s 1.  Ref: 11052668 Biochemistry, 2000
- Finally, our results indicate that the  **CheY**  surfaces that bind  **CheA** , FliM, and CheZ overlap, but are not completely identical.  Ref: 9295320 J Biol Chem, 1997
- **CheY**  is phosphorylated on Asp57 by the sensor kinase  **CheA**  and dephosphorylated by CheZ.  Ref: 12591865 J Bacteriol, 2003
- Co localization of  **CheY**  and CheZ with MCPs was  **CheA**  dependent, and co localization of  **CheA**  with MCPs was CheW dependent, as expected.  Ref: 10972797 Mol Microbiol, 2000
- Reverse transcription PCR confirmed that the other downstream chemotaxis genes within the same operon cheW, cheX, and  **cheY**  were still expressed in the  **cheA**  mutant strain.  Ref: 12003957 J Bacteriol, 2002
- Binding of attractant ligand to its receptor inactivates a receptor associated histidine kinase,  **CheA** , which phosphorylates the signal protein  **CheY** .  Ref: 10500179 Proc Natl Acad Sci U S A, 1999
- **CheA**  receives signals from chemoreceptors and then transfers the signal to  **CheY**  by a phosphotransfer reaction.  Ref: 9295320 J Biol Chem, 1997
- It is controlled by changes in the phosphorylation level of the chemotactic response regulator,  **CheY** , mediated by a histidine kinase  **CheA**  and a specific phosphatase CheZ.  Ref: 9837737 J Mol Biol, 1998
- The histidine protein kinase  **CheA**  2 mediates an attractant normal response via  **CheY**  4, while  **CheA**  1 and  **CheY**  5 appear to mediate a repellent inverted response.  Ref: 10970853 EMBO J, 2000
- In comparing  **CheA**  mediated phosphorylation of  **CheY**  to small molecule mediated phosphorylation of  **CheY** , the large difference in kphos values suggests that phospho  **CheA**  makes significant contributions to the catalysis of  **CheY**  phosphorylation.  Ref: 10029518 Biochemistry, 1999
- Functional domains of the T. denticola  **CheA**  and  **CheY**  proteins are highly conserved with those of the Escherichia coli Ec  **CheA**  and  **CheY**  proteins.  Ref: 10333522 Gene, 1999
- The level of P approximately  **CheY**  is controlled by the activity of the kinase  **CheA** , which is in turn regulated by membrane receptors at the cell surface.  Ref: 10207912 Novartis Found Symp, 1999
- In vitro,  **CheY**  can be phosphorylated either by phosphotransfer from phospho  **CheA**  or by acquiring a phosphoryl group from any of a variety of small, high energy phosphodonor molecules such as acetyl phosphate.  Ref: 10029518 Biochemistry, 1999
- **CheA**  passes its phosphate to a small, single domain response regulator,  **CheY** .  Ref: 10500847 Adv Microb Physiol, 1999
- Here, we report the crystal structure, at 2.95 A resolution, of the response regulator of bacterial chemotaxis,  **CheY** , bound to the recognition domain from its cognate histidine kinase,  **CheA** .  Ref: 9437425 Nat Struct Biol, 1998
- The first gene of the operon cheAY codes for a large 108 kDa polypeptide with an amino terminal domain that is homologous to  **CheA**  and a carboxyl terminus that is homologous to  **CheY** . cheAY is followed by cheW, an additional homolog of  **cheY** , cheB, and cheR.  Ref: 9294426 J Bacteriol, 1997
- The chemotaxis response regulator  **CheY**  can acquire phosphoryl groups either from its associated autophosphorylating protein kinase,  **CheA** , or from small phosphodonor molecules such as acetyl phosphate.  Ref: 10471801 FEBS Lett, 1999
- The protein  **CheY**  shuttles between flagellar motors and methyl accepting chemoreceptor MCP complexes containing the linker CheW and the kinase  **CheA** .  Ref: 11243808 J Mol Biol, 2001
- Scrutiny of the 26695 genome sequence suggests that H. pylori has two  **CheY**  response regulators one a separate protein CheY1 and the other CheY2 fused to the histidine kinase sensor  **CheA** .  Ref: 10722597 Infect Immun, 2000
- , with  **cheA**  to cheZ deleted with a low  **CheY**  level but a high cytoplasmic fumarate concentration displayed the same correlation of switching frequency and bias as cells overexpressing  **CheY**  at the wild type fumarate level.  Ref: 9642190 J Bacteriol, 1998
- Here we use fluorescence resonance energy transfer to monitor interactions of  **CheY**  approximately P with its phosphatase, CheZ, that reveal changes in the activity of the receptor kinase,  **CheA** , resulting from the addition of attractants or repellents.  Ref: 11742065 Proc Natl Acad Sci U S A, 2002
- **CheY** , the response regulator of bacterial chemotaxis, plays a pivotal role in signal transduction in bacterial chemotaxis and interacts with at least three proteins  **CheA** , FliM, and CheZ.  Ref: 9295320 J Biol Chem, 1997
- Binding of an attractant to the chemoreceptors inhibits  **CheA** , which results in decreased phosphorylation of the chemotaxis response regulator  **CheY** .  Ref: 12119291 J Biol Chem, 2002
- A truncated  **CheA**  construct that lacks the phosphoaccepting H domain and the  **CheY**  CheB binding domain forms an activated ternary complex that is similar to the one formed by the full length  **CheA**  protein.  Ref: 10350484 Biochemistry, 1999
- ...  **CheY**  provides catalytic functions for the phosphotransfer reaction, the  **CheA**  kinase may act simply to increase the effective phosphodonor concentration at the  **CheY**  active site.  Ref: 10471801 FEBS Lett, 1999
- The active site of  **CheY**  is exposed by the binding of the kinase domain, possibly to enhance phosphotransfer from  **CheA**  to  **CheY** .  Ref: 9636149 Proc Natl Acad Sci U S A, 1998

**Non-interactive relationship** (e.g. studied together, co-existance, homology, etc.)

- This is in direct contrast to the enterics in which  **CheA** , CheW and  **CheY**  are absolutely essential for taxis to PTS sugars, oxygen and MCP dependent chemoeffectors.  Ref: 9426144 Mol Microbiol, 1997
- Although phosphotransfer was slower with CheADeltaP2 k cat K m approximately 1.5 x 10 6 M 1 s 1 than with wild type  **CheA**  k cat K m approximately 10 8 M 1 s 1, it was still orders of magnitude faster than the kinetics of  **CheY**  phosphorylation by phosphoimidazole and other small molecule phosphodonors k cat K m approximately 5 50 M 1 s 1.  Ref: 11052668 Biochemistry, 2000
- For  **CheA**  > **CheY**  phosphotransfer, increasing ionic strength resulted in increased Ks values while kphos was unaffected.  Ref: 10029518 Biochemistry, 1999
- The third locus encodes two  **CheA** , one CheR, one CheB, one CheW, one  **CheY** , a putative cytoplasmic chemoreceptor TlpT and a protein showing homology to the chromosomal partitioning factor Soj designated Slp.  Ref: 12421313 Mol Microbiol, 2002
- Further insights into the mechanism of function of the response regulator  **CheY**  from crystallographic studies of the  **CheY**   **CheA**  124 257 complex.  Ref: 11134926 Acta Crystallogr D Biol Crystallogr, 2001
- It is difficult to study this interaction in vivo, because the dynamics of phosphorylation of  **CheY**  by its kinase  **CheA**  and the hydrolysis of  **CheY**  accelerated by CheZ are not under direct experimental control.  Ref: 9419353 Proc Natl Acad Sci U S A, 1998
- Previous work explored the rapid kinetics of  **CheY**  phosphorylation by  **CheA** .  Ref: 10029518 Biochemistry, 1999
- Reverse transcriptase polymerase chain reaction RT PCR data indicated that  **cheA**  through  **cheY**  are co transcribed and suggested that transcription is terminated after  **cheY** .  Ref: 10333522 Gene, 1999
- New crystallographic structures of the response regulator  **CheY**  in association with  **CheA**  124 257, its binding domain in the kinase  **CheA** , have been determined.  Ref: 11134926 Acta Crystallogr D Biol Crystallogr, 2001
- Signal transduction occurs via a phosphotransfer pathway involving a histidine protein kinase,  **CheA** , and a response regulator protein,  **CheY** , that in its phosphorylated state, modulates the direction of flagellar rotation.  Ref: 10049806 J Struct Biol, 1998
- Although P. aeruginosa was found to have proteins similar to the enteric chemotaxis proteins  **CheA** , CheB, CheW,  **CheY** , and CheZ, the gene encoding a CheR homologue did not reside in the chemotaxis gene cluster.  Ref: 10052136 Biosci Biotechnol Biochem, 1999
- Rhodobacter sphaeroides has multiple copies of chemotaxis genes two  **cheA** , one cheB, two cheR, three cheW, five  **cheY**  but no cheZ, controlling a single stop start flagellum.  Ref: 10970853 EMBO J, 2000
- The Td che genes have the same transcriptional orientation with a sigma 70 like promoter located upstream of  **cheA**  and a stem loop structure characteristic of a Rho independent transcriptional terminator downstream of  **cheY** .  Ref: 10333522 Gene, 1999
- This study, as anticipated from in vivo experiments, demonstrates in vitro that an attractant bound chemoreceptor leads to an increase in  **CheA**  activity, which in turn leads to an increase in the  **CheY**  P pool that ultimately causes a behavioural change in the bacteria.  Ref: 12094812 Microbiology, 1997
- Asparagine has been found to increase the rate of  **CheY**  P formation in the presence of McpB containing membranes,  **CheA** , and an excess of  **CheY** .  Ref: 12094812 Microbiology, 1997
- The gene cluster contained three chemotaxis che genes  **cheA** , cheW, and  **cheY**  and an open reading frame cheX that is homologous with Treponema pallidum Tp and Borrelia burgdorferi Bb cheX.  Ref: 10333522 Gene, 1999
- The nucleotide sequence of a region downstream of the Listeria monocytogenes flagellin gene, flaA, revealed two putative chemotaxis genes,  **cheY**  and  **cheA** .  Ref: 10528396 Can J Microbiol, 1999
- For example, building up the pathway by adding back  **CheA**  and  **CheY**  to a gutted strain lacking chemotaxis genes resulted in counterclockwise flagellar rotation whereas breaking down the pathway by deleting chemotaxis genes except  **cheA**  and  **cheY**  resulted in alternating episodes of clockwise and counterclockwise flagellar rotation.  Ref: 9683468 J Bacteriol, 1998
- This paper focuses on structural characteristics of the four signaling proteins  **CheA** ,  **CheY** , CheB, and CheR for which NMR or x ray crystal structures have been determined.  Ref: 10049806 J Struct Biol, 1998
- Deletion of either the complete chemosensory operons 1 and 2 plus the response regulator genes  **cheY**  4 and  **cheY**  5 or  **cheA**  2 alone led to the loss of all aerotactic responses, although the cells still swam normally.  Ref: 12270816 J Bacteriol, 2002
- Kinetic characterization of  **CheY**  phosphorylation reactions comparison of P  **CheA**  and small molecule phosphodonors.  Ref: 10029518 Biochemistry, 1999
- These facts provide evidence for a single pathway in which the PTS chemotactic signal is relayed rapidly to MCP CheW  **CheA**  signaling complexes that effect subsequent amplification and slower  **CheY**  dephosphorylation.  Ref: 10198062 Mol Biol Cell, 1999
- The cytoplasmic proteins  **CheY** , CheZ and  **CheA**  tended to cluster at the cell poles in a manner similar to that observed earlier for methyl accepting chemotaxis proteins MCPs, but only if MCPs were present.  Ref: 10972797 Mol Microbiol, 2000
- We used rapid quench and stopped flow fluorescence experiments to monitor phosphotransfer to  **CheY**  from phosphorylated wild type  **CheA**  and from phosphorylated CheADeltaP2.  Ref: 11052668 Biochemistry, 2000
- **CheA**  2 phosphorylated all of the CheYs and both CheBs, whilst  **CheA**  1 did not phosphorylate either CheB and phosphorylated only the response regulators encoded within its own genetic locus  **CheY**  1,  **CheY**  2, and  **CheY**  5 and  **CheY**  3.  Ref: 12421557 J Mol Biol, 2002
- The purple photosynthetic bacterium Rhodobacter sphaeroides has three loci encoding multiple homologues of the bacterial chemosensory proteins 13 putative chemoreceptors, four CheW, four  **CheA** , six  **CheY** , two CheB and three CheR.  Ref: 12421313 Mol Microbiol, 2002
- Rapid phosphotransfer to  **CheY**  from a  **CheA**  protein lacking the  **CheY**  binding domain.  Ref: 11052668 Biochemistry, 2000
- Characterization of the Tn5 insertion in JPA203 identified a second chemotaxis operon in R. sphaeroides that contains homologues of  **cheY** ,  **cheA**  and cheR, the first homologue of cheB and two homologues of cheW.  Ref: 9426144 Mol Microbiol, 1997
- Signal transduction in bacterial chemotaxis is mediated by a two component system composed of a protein histidine kinase,  **CheA** , and a response regulator,  **CheY** .  Ref: 10207912 Novartis Found Symp, 1999
- By contrast, under identical conditions, phosphorylation of  **CheY**  by phospho  **CheA**  occurs much more rapidly kphos approximately 800 s 1 and reflects  **CheY**  binding to phospho  **CheA**  considerably more tightly Ks approximately 60 microM than it does to the small molecule phosphodonors.  Ref: 10029518 Biochemistry, 1999
- To explore the contribution of P2 to the  **CheA**  >  **CheY**  phosphotransfer reaction in the Escherichia coli chemotaxis system, we examined the transfer kinetics of a mutant  **CheA**  protein CheADeltaP2 in which the 98 amino acid P2 domain had been replaced with an 11 amino acid linker.  Ref: 11052668 Biochemistry, 2000
- Although a chemotaxis operon has been identified containing homologues of the enteric  **cheA** , cheW, cheR genes and two homologues of the  **cheY**  gene, deletion of the entire chemotaxis operon had only minor effects on chemotactic behaviour under the conditions tested.  Ref: 9426144 Mol Microbiol, 1997
- Helicobacter pylori possesses two  **CheY**  response regulators and a histidine kinase sensor,  **CheA** , which are essential for chemotaxis and colonization of the gastric mucosa.  Ref: 10722597 Infect Immun, 2000
- Rhodobacter sphaeroides has multiple copies of these proteins 2 x  **CheA** , 3 x CheW and 3 x  **CheY** , but no CheZ.  Ref: 10632881 Mol Microbiol, 2000
- In bacteria, these receptors include the transmembrane sensor kinases, which control gene expression via their cognate response regulators, and chemoreceptors, which control the direction of flagellar rotation via the  **CheA**  kinase and  **CheY**  response regulator.  Ref: 11994152 Mol Microbiol, 2002
- The objective of this study was to probe the conformational coupling between the phosphorylation site and the signaling surface of  **CheY**  in the reverse direction by quantifying phosphorylation activity in the presence and absence of peptides of  **CheA** , CheZ, and FliM that specifically interact with  **CheY** .  Ref: 11353835 Proc Natl Acad Sci U S A, 2001
- The  **cheA** , cheW, cheX, and  **cheY**  genes are 240 33 62, and 438nt long, respectively, and encode proteins with predicted molecular masses of 88. 9. 6.8, and 16.  Ref: 10333522 Gene, 1999
- Molecular cloning, DNA sequencing and sequence analysis indicate that the dif locus encodes a new set of chemotaxis homologues of the bacterial chemotaxis proteins MCPs methyl accepting chemotaxis proteins, CheW,  **CheY**  and  **CheA** .  Ref: 9988486 Mol Microbiol, 1998
- Complementation of the single transposon copy mutant PF16 with cloned  **cheY**  and  **cheA**  in trans partially restored microaerotaxis and swarming on soft agar.  Ref: 10528396 Can J Microbiol, 1999
- Past experiments have shown that  **CheA**  and  **CheY**  are required to generate smooth swimming signals in Bacillus subtilis chemotaxis.  Ref: 12094812 Microbiology, 1997
- It is strongly suggested that the positive phototactic response was mediated by a phytochrome like photoreceptor and  **CheA**   **CheY**  type signal transduction system.  Ref: 11134414 Plant Cell Physiol, 2000
- The rate of phosphotransfer from 32P  **CheA**  phosphate to wild type  **CheY**  also decreased markedly > 150 times between pH 7.5 and 10.  Ref: 9398221 Biochemistry, 1997
- These results indicate that binding of  **CheY**  to the P2 domain of  **CheA**  indeed contributes to the rapid kinetics of phosphotransfer.  Ref: 11052668 Biochemistry, 2000
- The new genes were labelled orf10,  **cheY**  III,  **cheA**  II cheW II, cheW III, cheR II, cheB and tlpC.  Ref: 9426144 Mol Microbiol, 1997
- No phosphoryl group attached to Asp57 of  **CheY**  is visible from the electron density, but the response regulator in the  **CheY**   **CheA**  124 257 complex may have undergone a phosphorylation dephosphorylation process.  Ref: 11134926 Acta Crystallogr D Biol Crystallogr, 2001
- The two component regulatory system  **CheA**   **CheY**  has been shown to play a major role in chemotaxis in other enteric bacteria.  Ref: 10722597 Infect Immun, 2000
- The  **CheA**  and FliM binding surfaces of  **CheY**  have been well studied, but characterization of the CheZ binding surface of  **CheY**  is incomplete.  Ref: 9295320 J Biol Chem, 1997
- Null mutants in a methylated chemotaxis protein homolog FrzCD and a  **CheA**   **CheY**  homolog FrzE moved up a PE gradient at a reduced rate.  Ref: 9751772 Proc Natl Acad Sci U S A< MedlineTA>Proc Natl Acad Sci U S A, 1997
- The significance of these effects is discussed in relation to the catalytic mechanism of  **CheY**  phosphorylation by phospho  **CheA**  and small molecule phosphodonors.  Ref: 10029518 Biochemistry, 1999
- While searching for chemotaxis genes in M. xanthus, we identified a third chemotaxis like gene cluster, the che3 cluster, encoding homologs to two methyl accepting chemotaxis proteins MCPs, a CheW, a hybrid  **CheA** , a CheB, a CheR, but no  **CheY** .  Ref: 12566562 Proc Natl Acad Sci U S A, 2003
- Structure of the  **CheY**  binding domain of histidine kinase  **CheA**  in complex with  **CheY** .  Ref: 9437425 Nat Struct Biol, 1998
- While it is known that  **CheA**  activity is regulated, via the receptors, by chemotactic stimuli, the input that may regulate  **CheY**  dephosphorylation by CheZ has not been found.  Ref: 9837737 J Mol Biol, 1998
- We prepared fusions of yellow fluorescent protein the YFP variant of green fluorescent protein GFP with the cytoplasmic chemotaxis proteins  **CheY** , CheZ and  **CheA**  and the flagellar motor protein FliM, and studied their localization in wild type and mutant cells of Escherichia coli.  Ref: 10972797 Mol Microbiol, 2000
- The crystal structure at 2.0 A resolution of the complex of the Escherichia coli chemotaxis response regulator  **CheY**  and the phosphoacceptor binding domain P2 of the kinase  **CheA**  is presented.  Ref: 9636149 Proc Natl Acad Sci U S A, 1998
- Chemotaxis of Escherichia coli toward phosphotransferase systems PTSs carbohydrates requires phosphoenolpyruvate dependent PTSs as well as the chemotaxis response regulator  **CheY**  and its kinase,  **CheA** .  Ref: 10198062 Mol Biol Cell, 1999
- Together, these results suggest that the binding of  **CheY**  to  **CheA**  124 257 generates a geometry of the active site that favours phosphorylation.  Ref: 11134926 Acta Crystallogr D Biol Crystallogr, 2001
- The genome sequence of Campylobacter jejuni NCTC 11168 reveals the presence of orthologues of the chemotaxis genes  **cheA** , cheW, cheV,  **cheY** , cheR and cheB, ten chemoreceptor genes and two aerotaxis genes.  Ref: 11912013 Trends Microbiol, 2002
